# What are the factors associated with alcohol, cigarette and marijuana use among adolescents in Africa? Evidence from the Global School-based Health Survey

**DOI:** 10.1136/bmjopen-2024-089096

**Published:** 2025-07-28

**Authors:** Retselisitsoe Pokothoane, Terefe Gelibo Argefa, Josiane Djiofack Tsague, Noreen Dadirai Mdege

**Affiliations:** 1 Research Unit on the Economics of Excisable Products (REEP), School of Economics, University of Cape Town, Cape Town, South Africa; 2 Development Gateway: An IREX Venture, Washington, District of Columbia, USA; 3 Clinical Research, University of Ottawa Heart Institute, University of Ottawa, Ottawa, Ontario, Canada; 4 Department of Health Sciences, University of York, York, UK; 5 Centre for Research in Health and Development, York, UK

**Keywords:** Adolescents, Substance misuse, Tobacco Use

## Abstract

**Abstract:**

**Objectives:**

To provide comprehensive estimates of the prevalence of psychoactive substance use, specifically alcohol, cigarettes and marijuana, and factors associated with their use among school-going adolescents in 25 African countries.

**Design and methods:**

We used a pooled cross-sectional dataset from the publicly available Global School-based Health Survey (GSHS) from 25 African countries. We used descriptive statistics to estimate the prevalence of alcohol, cigarette and marijuana use as well as their dual use among adolescents aged 11–16 years. Additionally, we used logistic regressions to model factors associated with the use of each substance, with adjusted Odds Ratios (aORs) and their 95% Confidence Intervals (CIs) as the measures of association.

**Setting and participants:**

The study focused on school-going adolescents aged 11–16 years in 25 African countries that have conducted the GSHS between 2003 and 2017.

**Outcome measures:**

The key outcome measure is the proportion of adolescents who have used a specific substance in the past 30 days. These substances include: (1) alcohol, (2) cigarettes, (3) marijuana, (4) alcohol and cigarettes, (5) cigarettes and marijuana and (6) alcohol and marijuana.

**Results:**

The prevalence of alcohol use among adolescents was 9.5% (95% CI 8.4% to 10.7%), that of cigarette smoking was 6.2% (95% CI 5.0% to 7.6%), and it was 3.4% (95% CI 2.7% to 4.2%) for marijuana. The prevalence of dual use of alcohol and cigarettes was 3.1% (95% CI 2.4% to 3.9%), that of alcohol and marijuana was 2.0% (95% CI 1.5% to 2.5%), and it was 1.4% (95% CI 1.1% to 1.8%) for cigarettes and marijuana. The prevalence of cigarette smoking was significantly higher among boys than girls. However, there was no statistically significant difference in the prevalence of alcohol or marijuana by sex. Having parents who smoke any tobacco products, being bullied, missing school without permission and experiencing sadness and hopelessness were positively associated with being a current user, irrespective of substance type.

**Conclusions:**

There is a need for comprehensive, current data on substance use among adolescents. Interventions that tackle bullying, reduce school absenteeism, build resilience against difficult situations and increase self-efficacy to resist the use of these substances have the potential to curb substance use among adolescents in Africa.

**STRENGTHS AND LIMITATIONS OF THIS STUDY**

- This study is one of the first to comprehensively explore substance use across all African countries that have conducted the GSHS.
- The study used independent logistic regressions for each of the substances’ single and dual use, accounting for individual and country-level factors as well as different GSHS years.
- A limitation is that some GSHS datasets are old and may not reflect the current situation in respective countries.

## Introduction

 Psychoactive substance use (hereafter referred to as substance use) is one of the primary risky behaviours among adolescents, and it negatively affects their health, both in the current and long-term periods.^1^ Globally, the common substances consumed during adolescence are alcohol, tobacco and marijuana (cannabis).^2^ Harmful alcohol consumption ranks among the primary risk factors associated with poor health, resulting in approximately 3 million deaths globally each year.^3^ Similarly, tobacco smoking is highly addictive and kills over 8 million individuals worldwide annually.^4^ Furthermore, marijuana is linked to mental health problems among adolescents, which may persist into adulthood^5^ and ultimately pose a threat to their human capital development.

The United Nations (UN) Sustainable Development Goals 3.5 require UN member states to strengthen the control and prevention of substance use to promote good health and well-being for all individuals across the lifespan.^6^ Given that substance use typically begins during adolescence,^1^ it is crucial to understand the prevalence of use in this demographic group in order to implement effective substance use control measures. In Africa, although substance use remains a major public health issue, the prevalence of various substances is poorly documented.^7^ Among the member states of the World Health Organization (WHO), the Global School-based Health Survey (GSHS) serves as the primary data source for the surveillance of substance use beyond tobacco among adolescents. However, the existing cross-country analyses and syntheses of the GSHS data sets have been limited to a few African countries (8,^5 8^ 4^9^ and 23).^2^ Furthermore, these analyses are restricted to specific age groups that often exclude early adolescents (particularly those aged 11–12 years), as well as focusing on particular substances.

For the first time, we provide more comprehensive estimates of the prevalence of cigarettes, alcohol and marijuana, as well as their dual use (ie, current use of alcohol and cigarettes; alcohol and marijuana or cigarettes and marijuana), covering 25 African countries that have conducted at least one wave of GSHS. We included two additional countries, Sierra Leone and Seychelles, which were not part of any previous cross-country studies of the use of different substances. The age range available in the datasets varied across these 25 countries, from 11 to 18 years. For this study, we focused on the age group of 11–16 years, as this was the common range across the datasets, enabling us to include early adolescents. We also assessed the factors at both the country and individual levels that are associated with current use of these substances, including their dual use.

## Methods

### Data sources

This study used the most recent waves of the GSHS data from 25 African countries that have conducted at least one round of the survey. The datasets cover the period from 2003 to 2017. The GSHS is a school-based survey targeting adolescents in grades that correspond with the age group, 13–17 years, in WHO member states. Nonetheless, in many countries, the GSHS surveys tend to include adolescents aged 11–18 years. The overall objective of the survey is to collect information on the leading causes of morbidity and mortality among adolescents, including substance use. The survey was developed by the WHO in collaboration with the Centers for Disease Control and Prevention (CDC). The GSHS is a two-stage national cluster sample survey in which a sample of schools is selected in the first stage. In the second stage, classes are selected randomly within the selected schools, and all students within the selected classrooms participate by completing a self-administered questionnaire.^10^

Most of the 25 African GSHS datasets included participants aged 11–16 years. However, eight countries: Benin, Eswatini, Ghana, Liberia, Morocco, Mozambique, Mauritius and Namibia also included participants aged 17–18 years. To ensure uniformity across all surveys, we excluded any participants outside the 11–16 years age group from our analysis.

### Patient and public involvement

Patients or the public were not involved in the design, conduct, reporting or dissemination plans of our study.

### Substance use variables

Outcome variables were based on questions asked in GSHS and defined as follows:

Current alcohol use: Consuming at least one standard drink containing alcohol for at least 1 day in the past 30 days before the survey. This was obtained from the question, “During the past 30 days, on how many days did you have at least a standard drink containing alcohol?” Those who reported at least 1 day were considered current alcohol drinkers, and those who did not report any drinking day were classified as current non-drinkers.

Current cigarette smoking: Smoking cigarettes for at least 1 day in the past 30 days preceding the survey. The participants were asked, “During the past 30 days, how many days did you use cigarettes?” Those who reported at least 1 day were classified as current cigarette smokers. Otherwise, they were classified as current non-cigarette smokers.

Current marijuana use: Using marijuana one or more times in the past 30 days before the survey. This was retrieved from the following survey question: “During the past 30 days, how many times did you use marijuana?” Those who reported using marijuana at least once in the past 30 days were categorised as current marijuana users, and those who reported not using marijuana in the past 30 days were classified as current non-marijuana users.

Current dual use of alcohol and cigarettes: Participants who reported using alcohol and cigarettes in the past 30 days were categorised as dual users of alcohol and cigarettes. Those who used none or only one of the products were classified as not current dual users of alcohol and cigarettes.

Current dual use of alcohol and marijuana: Participants who reported using alcohol and marijuana in the past 30 days were classified as current dual users of alcohol and marijuana. Those who reported using none of these products or just one of them but not both were classified as not current dual users of alcohol and marijuana.

Current dual use of cigarettes and marijuana: Participants who reported using both cigarettes and marijuana in the past 30 days were classified as dual cigarette and marijuana users. Those who reported using none of these substances or using only one of them were classified as not current dual users of cigarettes and marijuana.

### Other variables of interest

Our analysis used information from the literature^2 5 7 8^ and the GSHS questionnaires, incorporating both individual-level (demographic and interpersonal) and country-level (ie, geographic) variables, as indicated below.

#### Demographic variables

The demographic variables included in this study include gender, age, number of close friends, parental/guardian smoking status and current school level (primary/secondary).^2 5 7 8^ The current school level variable was constructed using the information on students’ current class grades. We could not use the ‘grade’ variable directly as it is because African countries have different education systems and grade designations. We selected the mentioned variables due to their documented associations with substance use in the literature. Because of data constraints, we could not capture other important factors, such as individual income (pocket money) and product prices.

#### Interpersonal factors

The following personal/interpersonal factors were chosen based on their association with the use of the products of interest from different studies^2 8^:

- Being sad and hopeless: This variable assessed students’ stress-oriented feelings. It is a dummy variable generated from the survey question, “During the past 12 months, did you ever feel so sad or hopeless almost every day for two weeks or more in a row that you stopped doing your usual activities?” The variable was coded as 1 if a student responded ‘yes’ and 0 if they responded ‘no’.
- Bullying: This variable represented the experience of being bullied among students. It is a dummy variable generated from the survey question, “During the past 30 days, on how many days were you bullied?” This variable was coded as 1 if a respondent reported at least 1 day of experiencing bullying and 0 otherwise.
- Missing school: This represented students’ unauthorised school absence. It is a dummy variable generated from the survey question, “During the past 30 days, on how many days did you miss classes or school without permission?” This variable was coded as 1 if a respondent reported at least 1 day of missing school without permission, and 0 otherwise.

#### Country-level variables

Geographic location, country wealth and religion have been found to be associated with substance use.^2 8^ These variables were used as follows:

- Africa region: This categorical variable classified African countries by region: East Africa, North Africa, Southern Africa and West Africa.^11^ The Central Africa region is not represented in this study since none of the countries in that region had GSHS at the time of writing this article.
- World Bank income group: This categorical variable classified countries into low-income, lower-middle-income and upper-middle-income groups using data from the 2023 World Bank development indicators.^12^
- Religion: This categorical variable represented the dominant religion for each country: Christian or Muslim (inclusive of Hindu). Following de la Torre-Luque *et al*,^2^ we obtained the data from the report ‘Future of World Religions: Population Growth Projections: 2010–2050’.^13^

### Data analysis

We used Stata Version 17^14^ and appended all 25 surveys to obtain the consolidated sample of 66 145 adolescents aged 11–16 years. We obtained pooled prevalence estimates of the three substances for all 25 countries. Prevalence estimates were computed for each country and by African region, World Bank income group, age group and gender. All prevalence estimates were reported with the 95% CIs. We used the sample weights to adjust the surveys for non-responses and sample representation in each country. Specifically, for the within-country prevalence estimates, we used the sample weights that come with each survey. We reweighted the data for the pooled and regional prevalences for two reasons. First, to account for the fact that countries have different adolescent populations. Second, relative to each country’s adolescent population, GSHS has ‘oversampled’ adolescents in some countries and ‘undersampled’ in others (see online supplemental table 1). Following Filby and Van Walbeek,^15^ we computed each country’s weight for pooled analysis as follows:


$${weight}_{i}=\frac{\sum_{i=1}^{i=25}n_{i}}{n_{i}}\times \frac{N_{i}}{\sum_{i=1}^{i=25}N_{i}}$$

Where $n_{i}$ is the sample size of the GSHS for country i, and $N_{i}$ is the current population of

adolescents aged 11–16 years for country i, obtained from the United Nations World

Population Prospects database.^16^ To obtain the final weights used in the pooled prevalence

rates, we combined (multiplied) the country weights with the survey weights.

Multivariate logistic regression analysis was used to identify the factors associated with the use of alcohol, cigarettes or marijuana, as well as dual alcohol and cigarettes use, dual alcohol and marijuana use, and dual cigarettes and marijuana use. We estimated six independent logistic regression models differing in terms of the dependent variables, but similar in terms of independent variables. We presented the aORs, their 95% CIs and the associated p values.

Our analysis (ie, using GSHS datasets from 2003 to 2017) includes data that may be considered outdated. To rule out the possibility that prevalence rates may have changed over time, we performed sensitivity analysis by excluding all surveys conducted before 2010.

## Results

### Sample characteristics

Table 1 presents the descriptive statistics. The mean age in the sample was 14.3 years (SD=2.6 years). The sample included more girls (53.3%) than boys (47.7%). Most of the students were enrolled in secondary schools (81.4%), and resided in African countries where Christianity is the dominant religion (52.8%). Most participants were from countries classified as lower-middle-income (63.7%). Each of the following regions: East Africa, North Africa and Southern Africa contributed nearly 30% to the sample. We did not identify any GSHS datasets from countries in Central Africa. The respondents in the GSHS predominantly came from the survey period 2003 to 2005, making up 30% of the sample. Close to half (46.3%) reported experiencing bullying on at least 1 day during the past 30 days. Additionally, 45.1% stated that they had missed a class without permission at least once during the same period, and 28.1% reported feeling sad and hopeless almost every day for two or more consecutive weeks in the past 12 months.

**Table 1:** Characteristics of the participants from the GSHS in 25 African countries

Variable	Number of observations (n)	%
All	66 145	100
Gender		
Boys	31 055	47.7
Girls	34 006	53.3
Age		
11 years	1374	2.1
12 years	4759	7.3
13 years	11 786	18.1
14 years	15 250	23.4
15 years	16 807	25.8
16 years	15 081	23.2
School level		
Primary	12 055	18.6
Secondary	52 933	81.4
Region		
East Africa	19 273	29.1
North Africa	19 614	29.7
Southern Africa	18 164	27.5
West Africa	9094	13.8
World Bank income group		
Upper middle income	12 223	18.5
Lower middle income	42 107	63.7
Low income	11 815	17.8
Predominant country religion		
Christian	34 921	52.8
Hindu	2538	3.8
Muslim	28 686	43.4
Survey year		
2003–2005	20 046	30.3
2006–2008	6889	10.4
2009–2011	11 522	17.4
2012–2014	12 084	18.3
2015–2017	15 604	23.6
Bullying		
Yes	24 663	46.3
No	28 618	53.7
Missed classes without permission		
Yes	26 997	45.1
No	32 837	54.9
Feeling sad and hopeless		
Yes	13 609	28.1
No	34 852	71.9

Since there is only one high income country in the GSHS, it is grouped together with upper middle income countries. The number of observations (n) and percentages (%) are unweighted.

GSHS, Global School-based Health Survey.

### Prevalence of current use of alcohol, cigarettes and marijuana

Among the three substances, current alcohol drinking was more common, with a prevalence of 9.5% (95% CI 8.4% to 10.7%). This was followed by current cigarette smoking at 6.2% (95% CI 5.0% to 7.6%) and current marijuana use at 3.4% (95% CI 2.7% to 4.2%). The prevalence of dual use for alcohol and cigarettes was 3.1% (95% CI 2.4% to 3.9%) while that of alcohol and marijuana was 2.0% (95% CI 1.5% to 2.5%). Finally, the dual use of cigarettes and marijuana was 1.4% (95% CI 1.1% to 1.8%) (table 2).

**Table 2:** Prevalence of alcohol, cigarettes and marijuana use by country, region and income group

	Alcohol (95% CI)	Cigarettes (95% CI)	Marijuana (95% CI)	Dual alcohol and cigarette use (95% CI)	Dual alcohol and marijuana use (95% CI)	Dual cigarette and marijuana use (95% CI)
East Africa						
Djibouti (2007)	X	6.1% (4.8% to 7.8%)	X	X	X	X
Kenya (2003)	12.9% (9.1% to 18.1%)	16.1% (12.2% to 21.1%)	X	8.0% (4.6% to 13.5%)	X	X
Mauritius (2017)	23.4% (21.0% to 26.1%)	16.5% (13.7% to 19.8%)	5.2% (3.8% to 7.2%)	9.9% (8.2% to 12.0%)	3.7% (2.6% to 5.3%)	3.9% (2.7% to 5.5%)
Seychelles (2015)	47.6% (44.6% to 50.7%)	19.7% (17.4% to 22.2%)	9.3% (7.5% to 11.4%)	16.1% (13.9% to 18.5%)	6.9% (5.5% to 8.8%)	6.5% (5.1% to 8.2%)
Sudan (2012)	X	6.8% (5.1% to 8.9%)	X	X	X	X
Tanzania (2014)	4.6% (3.5% to 6.0%)	4.7% (3.6% to 6.1%)	2.6% (1.9% to 3.6%)	1.5% (0.9% to 2.3%)	1.4% (0.9% to 2.0%)	0.9% (0.6% to 1.4%)
Uganda (2003)	10.7% (9.1% to 12.4%)	4.9% (3.8% to 6.4%)	X	2.4% (1.8% to 3.2%)	X	X
Regional prevalence	6.9% (5.5% to 8.7%)	7.4% (6.0% to 9.0%)	2.6% (1.9% to 3.6%)	3.0% (2.0% to 4.5%)	1.4% (0.9% to 2.0%)	0.9% (0.6% to 1.6%)
North Africa						
Algeria (2011)	X	9.5% (7.8% to 11.6%)	2.2% (1.5% to 3.2%)	X	X	1.2% (0.7% to 2.1%)
Egypt (2011)	X	5.2% (3.1% to 8.6%)	X	X	X	X
Libya (2007)	X	4.0% (3.3% to 4.9%)	X	X	X	X
Mauritania (2010)	X	17.5% (13.8% to 22.1%)	6.9% (3.9% to 12.1%)	X	X	4.9% (2.6% to 8.8%)
Morocco (2016)	X	5.9% (5.0% to 7.0%)	5.4% (4.1% to 7.1%)	X	X	2.1% (1.6% to 2.9%)
Tunisia (2008)	X	8.3% (7.0% to 9.8%)	X	X	X	X
Regional prevalence	X	5.6% (3.8% to 8.3%)	3.5% (2.8% to 4.4%)	X	X	1.6% (1.2% to 2.1%)
Southern Africa						
Botswana (2005)	22.5% (20.1% to 25.0%)	7.8% (6.7% to 9.0%)	X	5.8% (4.8% to 6.9%)	X	X
Eswatini (2013)	X	X	3.1% (2.1% to 4.3%)	X	X	X
Malawi (2009)	4.2% (2.0% to 8.6%)	4.7% (3.1% to 7.1%)	X	1.6% (0.7% to 3.2%)	X	X
Mozambique (2015)	10.2% (7.0% to 14.7%)	2.2% (0.9% to 5.0%)	1.4% (0.7% to 2.6%)	0.9% (0.4% to 1.9%)	1.1% (0.4% to 2.7%)	0.3% (0.1% to 1.2%)
Namibia (2013)	26.9% (24.7% to 29.2%)	8.7% (7.1% to 10.7%)	5.1% (3.8% to 6.7%)	6.1% (5.0% to 7.4%)	3.2% (2.4% to 4.4%)	2.7% (1.9% to 3.9%)
Zambia (2004)	27.1% (24.3% to 30.0%)	X	X	X	X	X
Zimbabwe (2003)	14.4% (11.8% to 17.5%)	9.9% (8.7% to 11.3%)	X	5.3% (4.2% to 6.7%)	X	X
Regional Prevalence	10.5% (8.0% to 13.6%)	3.1% (1.9% to 4.8%)	1.4% (0.7% to 2.6%)	1.3% (0.8% to 2.0%)	1.1% (0.4% to 2.7%)	0.4% (0.1% to 1.1%)
West Africa						
Benin (2016)	40.0% (34.7% to 45.4%)	4.2% (2.7% to 6.4%)	1.0% (0.4% to 2.4%)	4.3% (2.8% to 6.4%)	0.9% (0.3% to 2.7%)	0.5% (0.2% to 1.4%)
Ghana (2012)	14.5% (12.4% to 17.0%)	8.5% (5.7% to 12.4%)	7.4% (5.1% to 10.5%)	5.2% (3.5% to 7.6%)	4.6% (3.3% to 6.4%)	3.3% (1.9% to 5.6%)
Liberia (2017)	18.4% (15.5% to 21.7%)	7.4% (5.5% to 9.8%)	6.3% (4.0% to 9.7%)	3.1% (1.9% to 4.9%)	3.0% (1.9% to 4.8%)	3.1% (1.9% to 4.8%)
Senegal (2005)	5.7% (3.3% to 9.9%)	8.5% (4.7% to 15.0%)	X	2.9% (1.4% to 5.8%)	X	X
Sierra Leone (2017)	11.9% (8.8% to 15.8%)	X	4.2% (2.7% to 6.6%)	X	2.6% (1.5% to 4.6%)	X
Regional Prevalence	16.3% (13.9% to 19.1%)	8.0% (6.0% to 10.5%)	6.4% (4.6% to 8.9%)	4.8% (3.5% to 6.6%)	4.0% (3.0% to 5.5%)	2.9% (1.9% to 4.6%)
WB income group						
Upper middle income	24.9% (23.2% to 26.7%)	4.6% (3.8% to 5.7%)	5.1% (3.9% to 6.5%)	6.3% (5.3% to 7.4%)	3.3% (2.5% to 4.3%)	2.8% (2.0% to 4.0%)
Lower middle income	9.5% (8.3% to 10.9%)	6.5% (5.1% to 8.1%)	3.7% (3.0% to 4.5%)	3.5% (2.7% to 4.6%)	2.1% (1.7% to 2.8%)	1.5% (1.2% to 2.0%)
Low income	9.3% (7.3% to 11.8%)	4.6% (3.6% to 5.9%)	1.6% (1.0% to 2.7%)	1.5% (1.0% to 2.1%)	1.2% (2.5% to 4.3%)	0.4% (0.2% to 1.1%)
Overall prevalence	9.5% (8.4% to 10.7%)	6.2% (5.0% to 7.6%)	3.4% (2.7% to 4.2%)	3.1% (2.4% to 3.9%)	2.0% (1.5% to 2.5%)	1.4% (1.1% to 1.8%)

X indicates that the data were not available.

WB, World Bank.

**Figure 1:**
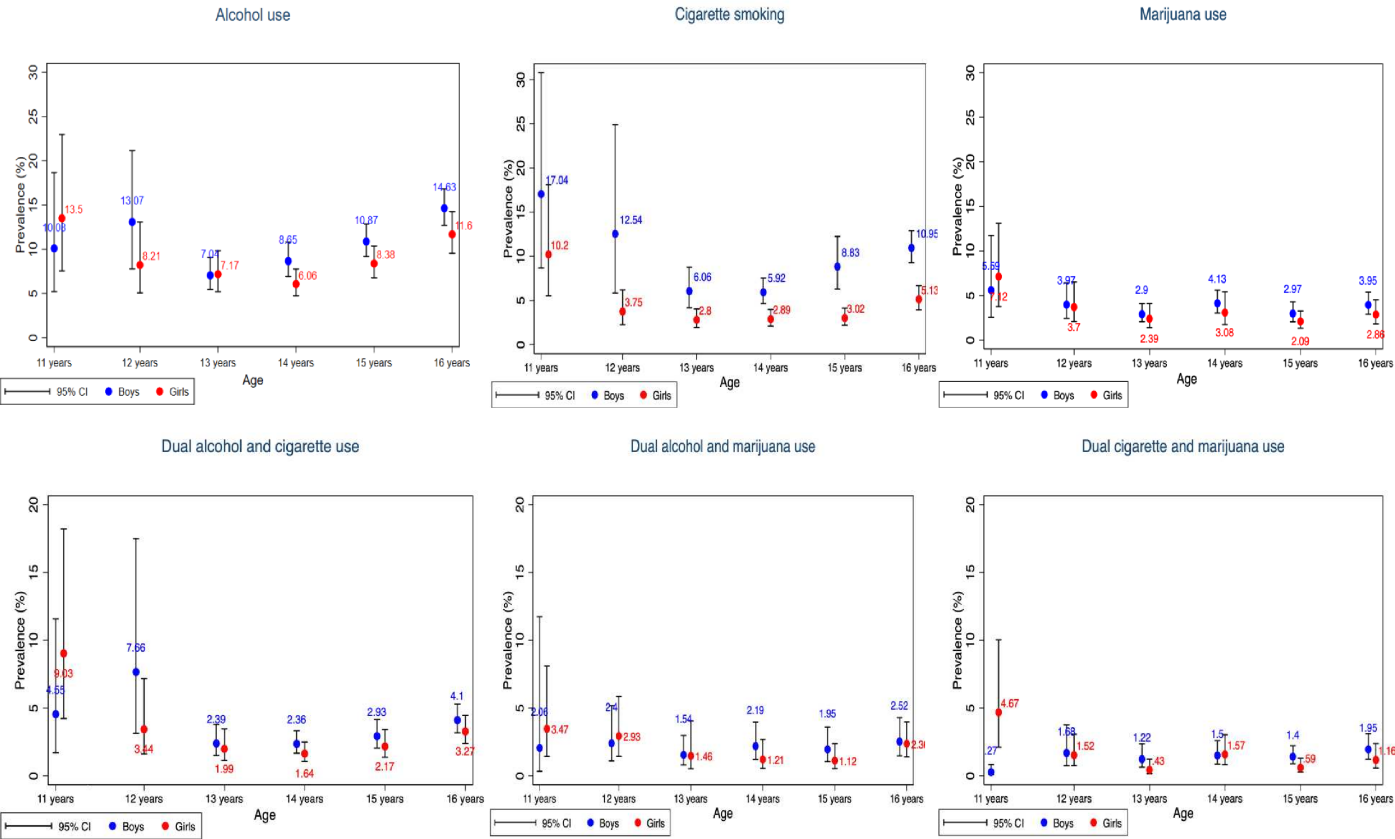
Prevalence of alcohol, cigarette and marijuana use among adolescents by age and gender.

Figure 1 shows the prevalence of cigarette, alcohol and marijuana use by gender and age. Among adolescents aged 13–16 years, there was a statistically significant difference in cigarette smoking prevalence by gender. However, there was no significant difference for those aged 11-12 years. Regarding the prevalence of alcohol and marijuana use, there was no statistically significant difference by gender for all age groups.

Regionally, the prevalence of alcohol drinking was 16.3% (95% CI 13.9% to 19.1%) for West Africa, 10.5% (95% CI 8.0% to 13.6%) for Southern Africa, and 6.9% (95% CI 5.5% to 8.7%) for East Africa. At the country level, the prevalence of alcohol drinking ranged from 4.2% in Malawi to 47.6% in Seychelles (table 2). The prevalence of alcohol consumption tends to increase with the country’s income group. Furthermore, alcohol consumption was insignificantly higher among boys (10.6% (95% CI 9.4% to 11.9%)) compared with girls (8.3% (95% CI 7.1% to 9.7%)) (online supplemental table 3).

The prevalence of cigarette smoking was similar in East Africa (7.4% (95% CI 6.0% to 9.0%)), North Africa (5.6% (95% CI 3.8% to 8.3%)) and West Africa (8.0% (95% CI 6.0% to 10.5%)). The prevalence was significantly lower in Southern Africa (3.1% (95% CI 1.9% to 4.8%)) (table 2). Cigarette smoking prevalence varied significantly by country. It was highest in Seychelles at 19.7% (95% CI 17.4% to 22.2%) and lowest in Mozambique at 2.2% (95% CI 0.9% to 5.0%) (table 2). Additionally, cigarette smoking increased with countries’ World Bank income group. Boys reported a higher cigarette smoking prevalence (8.6% (95% CI 6.8% to 11.0%)) than girls (3.5% (95% CI 2.8% to 4.3%)) (online supplemental table 3).

The overall prevalence of marijuana was highest in West Africa, 6.4% (95% CI 4.6% to 8.9%), and lowest in Southern Africa, 1.4% (95% CI 0.7% to 2.6%) (table 2). This prevalence was insignificantly higher among boys, 3.6% (95% CI 3.0% to 4.4%) compared with girls, 3.0% (95% CI 2.2% to 4.4%) (online supplemental table 3).

The dual use of alcohol and cigarettes, as well as alcohol and marijuana, was found to be similar and more prevalent than that of cigarettes and marijuana. West Africa had the highest prevalence of dual use among all the assessed products. At the country level, the prevalence of dual use of alcohol and cigarettes ranged from 0.9% (95% CI 0.4% to 1.9%) in Mozambique to 16.1% (95% CI 13.9% to 18.5%) in Seychelles. For alcohol and marijuana, the prevalence rates ranged from 0.9% (95% CI 0.3% to 2.7%) in Benin to 6.9% (95% CI 5.5% to 8.8%) in Seychelles. When considering the use of both cigarettes and marijuana, the prevalence ranged from 0.3% (95% CI 0.1% to 1.2%) in Mozambique to 6.5% (95% CI 5.1% to 8.2%) in Seychelles (table 2). The prevalence of dual substance use was higher among boys than girls (online supplemental table 4).

We did not find any significant differences in prevalence rates based on substance, gender, African region and World Bank income group (see online supplemental table 5), between the primary analysis and sensitivity analysis, which excluded surveys conducted before 2010. Thus, our prevalence rates remain consistent even when excluding datasets that may be considered outdated.

### Factors associated with the current use of alcohol, cigarettes and marijuana

Table 3 presents the association of different variables of interest with the odds of substance use, after mutually adjusting for the effects of other variables. In other words, we present the aOR of different variables in relation to the odds of substance use. Students living in West Africa were more likely to be current alcohol users, with an aOR of 2.6 (95% CI 1.7 to 3.1) compared with those from East Africa. Those from countries in the upper-middle World Bank income group had higher odds of being current alcohol users (aOR 3.2 (95% CI 1.4 to 6.9)) than those from low-income countries.

**Table 3:** Logistic regression results of the factors associated with the current use of different substances

Variables	Alcohol (1)	Cigarettes (2)	Marijuana (3)
	aOR (95% CI)	aOR (95% CI)	aOR (95% CI)
Age	1.1 (1.0, 1.2)	1.0 (0.9, 1.1)	1.0 (0.8, 1.2)
	[0.097]	[0.991]	[0.756]
Girl (Ref: Boy)	0.9 (0.8, 1.0)	0.5 (0.4, 0.7)	0.6 (0.3, 1.2)
	[0.043]	[0.000]	[0.178]
Education (Ref: Primary)			
Secondary	1.0 (0.8, 1.4)	0.7 (0.6, 0.9)	0.5 (0.4, 0.7)
	[0.836]	[0.021]	[0.000]
Africa region (Ref: East Africa)			
North Africa	X	1.0 (0.5, 1.8)	0.8 (0.6, 1.1)
	X	[0.945]	[0.191]
Southern Africa	1.5 (1.0, 2.2)	0.9 (0.7, 1.1)	1.0 (0.8, 1.3)
	[0.057]	[0.316]	[0.994]
West Africa	2.6 (1.7, 3.9)	1.5 (1.1, 2.0)	3.5 (2.7, 4.5)
	[0.000]	[0.011]	[0.000]
World Bank income group (Ref: Low income)			
Lower middle income	0.8 (0.6, 1.2)	1.9 (1.4, 2.4)	2.7 (1.5, 5.0)
	[0.311]	[0.000]	[0.001]
Upper middle income	3.2 (1.4, 6.9)	3.1 (1.5, 5.9)	5.2 (3.6, 7.4)
	[0.004]	[0.001]	[0.000]
Religion (Ref: Christian)			
Muslim and Hindu	0.9 (0.3, 2.8)	3.2 (1.3, 7.6)	0.7 (0.4, 0.8)
	[0.977]	[0.010]	[0.000]
Having close friends (Ref: None)			
One	1.3 (0.9, 1.7)	1.0 (0.9, 1.2)	0.6 (0.4, 0.9)
	[0.189]	[0.822]	[0.026]
At least two	1.4 (1.0, 2.2)	1.0 (0.7, 1.4)	0.7 (0.3, 1.5)
	[0.097]	[0.907]	[0.318]
Parental smoking (Ref: No)	2.5 (2.0, 3.1)	3.2 (2.5, 4.0)	4.5 (3.6, 5.6)
	[0.000]	[0.000]	[0.000]
Bullying (Ref: No)	1.9 (1.4, 2.6)	2.9 (2.3, 3.6)	2.8 (2.0, 4.1)
	[0.000]	[0.000]	[0.000]
Sad and hopeless (Ref: No)	1.7 (1.5, 2.1)	2.1 (1.6, 2.8)	3.1 (2.1, 4.6)
	[0.000]	[0.000]	[0.000]
Missing school (Ref: No)	1.6 (1.1, 2.4)	1.9 (1.6, 2.3)	2.4 (1.8, 3.2)
	[0.016]	[0.000]	[0.000]
Observations	21 545	30 754	17 919

The p-values are in square brackets. The regression results are weighted, and the standard errors (SEs) are clustered at the country level. The time (survey years) effects are included in all three regressions. X indicates that the data were unavailable.

Additionally, students with two or more close friends were also more likely to be current alcohol users (aOR 1.4 (95% CI 1.0 to 2.2)) than those with no close friends. Those who had parents/guardians who used any tobacco products were more likely to be current alcohol users (aOR 2.5 (95% CI 2.0 to 3.1)) than those whose parents/guardians did not use tobacco. Those who had experienced bullying during the past 30 days (aOR 1.9 (95% CI 1.4 to 2.6)) were more likely to be current alcohol users compared with those who had not been bullied. Those who reported being sad and hopeless almost every day for 2 weeks or more in a row during the past 12 months were more likely to be current alcohol users (aOR 1.7 (95% CI 1.5 to 2.1)) compared with those who had not experienced sadness and hopelessness. Finally, students who missed school without permission at least once in the past 30 days were more likely to be current alcohol users (aOR 1.6 (95% CI 1.1 to 2.4)) than those who did not miss school (table 3, column 1).

For cigarette smoking, girls were less likely to be current smokers (aOR 0.5 (95% CI 0.4 to 0.7)) than boys. Students living in West Africa were more likely to be current cigarette smokers (aOR 1.5, 95% CI 1.1 to 2.0), compared with those in East Africa. Students from both lower-middle-income (aOR 1.9, 95% CI 1.4 to 2.9) and upper-middle-income countries (aOR 3.1, 95% CI (1.5 to 5.9)) had higher odds of being current cigarette smokers than those in low-income countries. Students from countries where Islam or Hinduism is the dominant religion were more likely to smoke cigarettes (aOR 3.2 (95% CI 1.3 to 7.6)) than those from countries where Christianity is the dominant religion (table 3, column 2).

Students whose parents used any tobacco products were more likely to be current cigarette smokers (aOR 3.2 (95% CI 2.5 to 4.0)) compared with those whose parents did not use tobacco. Those who experienced bullying in the past 30 days were more likely to be current cigarette smokers (aOR 2.9 (95% CI 2.3 to 3.6)) than those who did not. Furthermore, students who experienced feelings of sadness and hopelessness almost every day for two or more consecutive weeks during the past 12 months were more likely to be current cigarette smokers (aOR 2.1 (95% CI 1.6 to 2.8)) than those who did not. Those who missed school without permission for at least 1 day in the past 30 days were also more likely to be current cigarette smokers (aOR 1.9 (95% CI 1.6 to 2.3)) than those who did not miss school (table 3, column 2).

For marijuana use, students residing in West Africa were more likely to be current marijuana users (aOR 3.5 (95% CI 2.5 to 4.5)) than those from East Africa. Similarly, students from lower-middle-income countries had increased odds of current marijuana use (aOR 2.7 (95%CI: 1.5 to 5.0)), while those from upper-middle-income countries had even higher odds (aOR 5.2 (95% CI 3.6 to 7.4)) compared with their peers from low-income countries (table 3, column 3).

Students from countries where Islam or Hinduism are dominant religions were less likely to be current marijuana users (aOR 0.7 (95% CI 0.4 to 0.8)) than students from Christianity-dominated countries. Having one close friend was associated with lower odds of being a current marijuana user (aOR 0.6 (95% CI 0.4 to 0.9)) compared with having no close friends. Students whose parents/guardians used any tobacco products were more likely to be current marijuana users (aOR 4.5 (95% CI 3.6 to 5.6)) compared with those who did not. Those who reported being bullied in the past 30 days were more likely to be marijuana users (aOR 2.8 (95% CI 2.0 to 4.1)) compared with those who did not. Those who experienced feelings of sadness and hopelessness almost every day for 2 weeks or more consecutively in the past 12 months were more likely to be marijuana users (aOR 3.1 (95% CI 2.1 to 4.6) than those who did not. Lastly, those who missed school without permission for at least 1 day during the past 30 days were more likely to be current marijuana users (aOR 2.4 (95% CI 1.8 to 3.2)) than those who did not (table 3, column 3).

The dual use of substances, similar to single-use, shows that two location-related variables, residing in West Africa and living in upper-middle-income countries, were associated with the increased odds of being a current dual user for alcohol and cigarettes, alcohol and marijuana, and cigarettes and marijuana. Similarly, a number of interpersonal factors were also associated with increased odds of being a dual user. These factors include having parents who use tobacco products, being bullied in the past 30 days, experiencing sadness and hopelessness almost every day for 2 weeks or more in a row during the past 12 months, and missing school without permission for at least 1 day during the past 30 days (see table 4).

**Table 4:** Logistic regression results of the factors associated with the current dual use of different substances

Variables	Dual alcohol and cigarettes (1)	Dual alcohol and marijuana (2)	Dual cigarettes and marijuana (3)
	aOR (95% CI)	aOR (95% CI)	aOR (95% CI)
Age	1.0 (0.9, 1.2)	1.0 (0.7, 1.5)	1.0 (0.8, 1.3)
	[0.901]	[0.755]	[0.952]
Girl (Ref: Boy)	0.7 (0.6, 0.9)	0.8 (0.5, 1.2)	0.7 (0.3, 1.3)
	[0.003]	[0.273]	[0.266]
Education (Ref: Primary)			
Secondary	0.8 (0.4, 1.4)	0.5 (0.3, 0.8)	0.3 (0.1, 0.6)
	[0.400]	[0.004]	[0.002]
Africa Region (Ref: East Africa)			
North Africa	X	X	1.4 (0.8, 2.5)
	X	X	[0.200]
Southern Africa	1.0 (0.8, 1.3)	1.4 (0.4, 5.6)	0.5 (0.3, 1.1)
	[0.987]	[0.632]	[0.104]
West Africa	2.6 (2.0, 3.5)	3.9 (1.3, 11.6)	6.3 (1.9, 21.8)
	[0.000]	[0.014]	[0.003]
World Bank income group (Ref: Low Income)			
Lower middle income	1.8 (1.4, 2.3)	4.5 (0.5, 39.2)	2.8 (0.1,76.6)
	[0.019]	[0.170]	[0.538]
Upper middle income	5.1 (2.2,11.4)	7.4 (4.5, 25.5)	18.6 (4.3, 81.2)
	[0.000]	[0.002]	[0.000]
Religion (Ref: Christian)			
Muslim and Hindu	2.5 (1.0, 6.4)	0.6 (0.4, 1.0)	0.8 (0.6, 1.1)
	[0.048]	[0.061]	[0.213]
Having close friends (Ref: None)			
One	1.1 (0.8, 1.4)	0.6 (0.2, 1.6)	1.0 (0.8, 1.6)
	[0.718]	[0.264]	[0.597]
At least two	1.2 (0.8, 1.8)	1.1 (0.5, 2.4)	1.3 (0.7, 2.6)
	[0.525]	[0.810]	[0.411]
Parental smoking (Ref: No)	4.0 (2.7, 5.9)	7.4 (6.7, 8.2)	6.7 (3.5,11.3)
	[0.000]	[0.000]	[0.000]
Bullying (Ref: No)	3.2 (2.2, 4.6)	4.0 (2.5, 6.2)	5.9 (2.6, 7.1)
	[0.000]	[0.000]	[0.000]
Sad and hopeless (Ref: No)	2.3 (1.5, 3.5)	2.8 (1.4, 5.5)	3.8 (1.8, 8.1)
	[0.000]	[0.003]	[0.000]
Missed school (Ref: No)	2.2 (1.7, 2.7)	2.4 (1.4, 4.3)	1.9 (1.2, 3.1)
	[0.000]	[0.000]	[0.007]
Observations	21 029	11 597	17 595

The p-values are in square brackets. The regression results are weighted, and the SEs are clustered at the country level. The time effects (survey years) are included in all three regressions. X indicates that the data were unavailable.

## Discussion

We found that the overall prevalence of alcohol use (9.5%) was the highest, followed by cigarette smoking (6.2%) and then marijuana use (3.4%). The co-consumption of alcohol and cigarettes was more prevalent than that of alcohol and marijuana, or cigarettes and marijuana. Regardless of the type of substance, residing in West Africa as well as in upper-middle-income countries, having parents who use tobacco products, experiencing bullying, feeling sad and hopeless, and missing school without permission were positively associated with being a current user of alcohol, cigarettes and marijuana. Other variables showed significant associations with only certain substances. For instance, being a girl was negatively associated with the use of alcohol and cigarettes. Additionally, predominant country-level religion and education level were significant only for cigarettes and marijuana use. Having close friends was positively associated with alcohol use but negatively associated with marijuana use.

Our finding that alcohol use is more prevalent than cigarette smoking or marijuana use aligns with other studies in Africa.^7^ Additionally, our estimates of alcohol prevalence estimates are consistent with previous studies that have used the GSHS.^2 9^ In European and Asian countries, the prevalence of alcohol use among school-going adolescents has also been found to be higher (20%) compared with 8% for cigarettes and 7% for marijuana.^17^ The significantly higher prevalence of alcohol use compared with cigarette smoking and marijuana use can be attributed to the fact that alcohol is generally more socially accepted in many cultures.^2^ In many countries, alcohol is also more available, accessible and cheaper than tobacco or marijuana use.^3^

Marijuana has the lowest prevalence (3.4%) among the three substances, potentially because it has long been considered illegal in all African countries and, therefore, might be the least socially acceptable substance. Some African countries, such as Lesotho and Zimbabwe, have legalised medical marijuana use (in 2017 and 2018, respectively), but recreational use remains illegal.^18^ It is important to monitor if this development will have an impact on the prevalence of its recreational use in the future.

Our current estimates for cigarette smoking prevalence are consistent with findings from a study of 53 African countries based on the Global Youth Tobacco Survey (GYTS) data. This study found that 6.4% (95% CI 5.9% to 7.0) of students aged between 11 and 17 years were current cigarette smokers.^19^ However, our estimates differ from a study conducted in 22 African countries using the GYTS and found the prevalence of 10.9% (95% CI 9.8% to 12.1%).^20^ This difference is potentially due to the fact that our study did not include adolescents aged 17, while the study of 22 countries did.

Our results also suggest a widening gender gap in current cigarette smoking behaviour between boys and girls as they age. This is different from what has been observed in Europe, Central Asia and Canada, where data suggest no significant difference in current smoking between boys and girls in most countries and regions.^21^ This discrepancy may reflect differences in the social acceptability of smoking among girls. In developed countries, this acceptability might be higher compared with countries in Africa. In Africa, as girls become more aware that smoking is generally considered less acceptable for them than for boys, it could result in fewer girls taking up smoking as they grow older.

The other study closely related to ours examines the prevalence of cigarette smoking and alcohol use, using GSHS data from nine countries in the African region and seven countries in the Americas for the years 2003–2007.^22^ This study primarily focuses on comparing the country-level prevalences of cigarette use, alcohol consumption and the dual use of both substances. In the nine African countries assessed, the prevalence of alcohol use ranged from 3.2% in Senegal to 61.6% in Seychelles, while the prevalence of cigarette smoking ranged from 3.8% in Tanzania to 17.2% in Seychelles. The dual prevalence of alcohol and cigarette use ranged from 1.5% in Tanzania to 15.8% in Seychelles. The results from this study differ from ours mainly because we included students aged 11–16 years, while their study only focused on students aged 13–15 years.^22^

Unlike our study, they did not present pooled prevalence across countries, making it impossible to conduct pooled and regional prevalence comparisons. Their study^22^ included nine African GSHS datasets that were conducted up to 2007, while ours included an additional 16 new datasets that were conducted up to 2017. Furthermore, we examined the factors associated with the use of various substances, while their study^22^ primarily focused on the prevalence of substance use.

Our estimates on marijuana prevalence align with the study of eight countries in sub-Saharan Africa, which found that the overall prevalence was between 4% and 5%,^5^ with Seychelles having the highest rate of marijuana use.^5 8^ Compared with Asian and European countries, marijuana prevalence among adolescents in African countries is relatively low.^23^ Our study also highlights the lack of data on monitoring marijuana and alcohol use among adolescents in African countries. While the GSHS serves as the primary data source for national-level alcohol and marijuana use prevalence estimates among young people in the WHO Member states, only 15 and 13 out of 54 African countries, respectively, have provided data on these substances (see online supplemental table 2).

Although we found a low prevalence of the co-consumption of substances, there is still a need for preventive interventions among adolescents. Adolescents who smoke and drink are at a higher risk of developing diseases related to smoking and drinking. They also have lower chances of quitting and are likely to continue using these substances in the long term.^24^ Additionally, those who co-consume cigarettes and alcohol also have an increased risk of having difficulties at school, delinquency^25^ and emotional development,^26^ than those who do not co-consume these products. The co-consumption of alcohol and marijuana substantially increases the likelihood of comorbid substance use and experiencing mental health disorders.^27^

An analysis of national-level data from eight sub-Saharan African countries found that experiencing bullying, sadness and hopelessness were positive factors of alcohol^8^ and marijuana use,^5^ but not cigarette smoking. This contrasts with our current study, where these variables were risk factors for all the substances. This difference could be due to our study including data from 17 more countries, thus providing a larger dataset, compared with the previous studies.^5 8^ Furthermore, evidence from 73 Low- and Middle-Income Countries (LMICs) also found that a country’s income group was an important factor only for marijuana smoking, while in our study this was important for all substances. This difference could be because in our study, we focused exclusively on African countries, while the 73-country study^2^ included both African and non-African countries. The measures of substance use used in the two studies varied. For example, we concentrated solely on current (past 30-day) use of the substances, while the 73-country study explored regular use and problematic use, particularly concerning alcohol.^2^

Consistent with other studies,^8^ being a girl was a protective factor against being a cigarette or alcohol user. The predominant religion (ie, Islam) at the country level was a risk factor for cigarette, marijuana and dual use of alcohol with cigarettes or marijuana, but not for alcohol use alone. This is in contrast with evidence from 73 LMICs,^2^ which suggested that the Islamic religion was a protective factor against alcohol use.

Young people are sensitive to increases in the prices of cigarettes^16^ and alcohol.^28^ One effective strategy that countries can adopt to reduce the use of the consumption of these substances is increasing their excise taxes. This could be complemented by strengthening restrictions on the availability and accessibility of the products, and comprehensive restrictions on their advertising, promotion and sponsorship.^3^ Research should develop and test whether interventions that reduce bullying behaviour, build resilience against difficult situations and increase self-efficacy among adolescents help prevent the initiation and continued use of these substances. Evidence from high-income countries like Iceland revealed that long-term community-based programmes that focus on reducing adolescents’ unsupervised time (ie, the time they spend alone) and increasing their participation in sports significantly reduced alcohol use in Iceland.^18^

To our knowledge, this is the most comprehensive study in Africa in terms of the number of countries included, the broader adolescent age group and the larger sample size. However, some of the datasets used are old, going as far back as 2003. Nonetheless, our sensitivity analysis excluding datasets before 2010 demonstrated the robustness of our estimates. Our analysis, therefore, provides the best available estimates, particularly for alcohol use and marijuana, where the only national-level data on adolescents is from the GSHS. It is important to note, however, that the results might not be generalised to all adolescents, as the GSHS does not include some groups, such as those who are out of school. There is evidence that suggests that out-of-school adolescents are more likely to use psychoactive substances than those who are in school,^26 29^ which means that our prevalence estimates may be lower than the reality.

Our logit regression models do not account for all factors that are theoretically and empirically associated with substance use because we rely on the data collected from the GSHS. For instance, factors such as price, pocket money and media-related factors have not been included in our analysis. There is well-established evidence that prices play a significant role in reducing the use of alcohol,^28^ cigarettes^16^ and marijuana.^30^ Our study highlights very limited monitoring of marijuana and alcohol use among adolescents in African countries. Future research should address these limitations to enhance our understanding and support better decision-making.

Our study provides prevalence estimates for alcohol use, cigarette smoking and marijuana use for Africa, both overall and at country and regional levels. It highlights the need for comprehensive and current data on substance use among adolescents. The study also calls for the development of interventions aimed at curbing substance use in this demographic group. These interventions should tackle issues such as bullying, school absenteeism, building resilience in difficult situations and increasing self-efficacy to resist the use of these substances.

## Supplementary material

10.1136/bmjopen-2024-089096 online supplemental file 1

## Data Availability

Data are available in a public, open access repository.
