# Supplementary material for: What are the factors associated with alcohol, cigarette and marijuana use among adolescents in Africa? Evidence from the Global School-based Health Survey
Source: BMJ Open. 2025 Jul 28;15(7):e089096. doi: 10.1136/bmjopen-2024-089096 (PMC12306306; doi:10.1136/bmjopen-2024-089096)
Supplement: online supplemental file 1 [file bmjopen-15-7-s001.pdf]

SUPPLEMENTARY INFORMATION

Supplementary Table 1. The population of adolescents aged 11-16 years and sample sizes for African countries with available GSHS datasets

Location	Latest adolescent population (N)	GSHS sample (n)
**East Africa**		
Djibouti (2007)	136 601	1 777
Kenya (2003)	8 162 155	3 691
Mauritius (2017)	91 211	2 538
Seychelles (2015)	9 832	2 514
Sudan (2012)	7 019 428	2 211
Tanzania (2014)	9 460 111	3 327
Uganda (2003)	7 438 139	3 215
**North Africa**		
Algeria (2011)	4 951 539	4 532
Egypt (2011)	13 840 546	2 568
Libya (2007)	837 882	2 242
Mauritania (2010)	735 918	2 063
Morocco (2016)	3 993 390	5 339
Tunisia (2008)	1 157 085	2 870
**Southern Africa**		
Botswana (2005)	293 612	2 197
Eswatini (2013)	159 517	1 975
Malawi (2009)	3 260 282	2 359
Mozambique (2015)	5 007 306	1 112
Namibia (2013)	368 214	2 732
Zambia (2004)	3 092 604	2 257
Zimbabwe (2003)	2 517 417	5 532
**West Africa**		
Benin (2016)	1 989 770	1 183
Ghana (2012)	4 460 272	1839
Liberia (2017)	813 908	988
Senegal (2005)	2 598 671	3 154
Sierra Leone (2017)	1 195 372	1930

Notes: The data on the adolescent population was obtained from the 2023 United Nations World Population Prospects database.

Supplementary Table 2. Latest data availability on the use of cigarettes, alcohol, and marijuana in African GSHS among adolescents, 2003 - 2017

Location	Cigarettes	Alcohol	Marijuana
**East Africa**			
Djibouti (2007)	✓	X	X
Kenya (2003)	✓	✓	X
Mauritius (2017)	✓	X	✓
Seychelles (2015)	✓	✓	✓
Sudan (2012)	✓	X	X
Tanzania (2014)	✓	✓	✓
Uganda (2003)	✓	✓	X
**Total available datasets**	**7**	**4**	**3**
**North Africa**			
Algeria (2011)	✓	X	✓
Egypt (2011)	✓	X	X
Libya (2007)	✓	X	X
Mauritania (2010)	✓	X	✓
Morocco (2016)	✓	X	✓
Tunisia (2008)	✓	X	X
**Total available datasets**	**6**	**0**	**3**
**Southern Africa**			
Botswana (2005)	✓	✓	X
Eswatini (2013)	X	X	✓
Malawi (2009)	✓	✓	X
Mozambique (2015)	✓	✓	✓
Namibia (2013)	✓	✓	✓
Zambia (2004)	X	✓	X
Zimbabwe (2003)	✓	✓	X
**Total available datasets**	**5**	**6**	**3**
**West Africa**			
Benin (2016)	✓	✓	✓
Ghana (2012)	✓	✓	✓
Liberia (2017)	✓	✓	✓
Senegal (2005)	✓	✓	X
Sierra Leone (2017)	X	✓	✓
**Regional Total**	**4**	**5**	**4**
**Total available GSHS in Africa**	**22**	**15**	**13**

Notes: ✓ indicates data availability, and X indicates data unavailability.

Supplementary Table 3. Prevalence of alcohol, cigarettes, and marijuana use: by country, region, and income group

	**Alcohol use**		**Cigarette smoking**		**Marijuana**	
	**Boys (95% CI)**	**Girls (95% CI)**	**Boys (95% CI)**	**Girls (95% CI)**	**Boys (95% CI)**	**Girls (95% CI)**
**East Africa**						
Djibouti (2007)	X	X	8.6% (6.3, 11.5)	2.6% (1.5, 4.6)	X	X
Kenya (2003)	14.7% (10.2, 20.8)	11.1% (7.1, 16.9)	19.3% (14.7, 24.9)	13.1% (9.0, 18.5)	X	X
Mauritius (2017)	24.6% (20.7, 29.0)	22.0% (17.1, 28.0)	22.4% (18.2, 27.2)	11.1% (7.7, 15.7)	9.6% 7.8, 11.9)	1.4% (0.8, 2.3)
Seychelles (2015)	47.1% (43.0, 51.2)	48.1% (44.6, 51.7)	24.5% (21.3, 27.9)	14.8% (12.4, 17.5)	13.3% (10.6, 16.7)	5.4% (4.0, 7.2)
Sudan (2012)	X	X	8.8% (6.9, 11.2)	4.5% (2.7, 7.5)	X	X
Tanzania (2014)	4.6% (3.4, 6.2)	4.1% (2.8, 5.9)	5.4% (3.9, 7.5)	3.6% (2.5, 5.2)	2.3% (1.4, .9)	2.6% (1.9, 3.6)
Uganda (2003)	9.2% (7.5, 11.1)	9.2% (7.5, 11.1)	6.8% (5.2, 8.8)	2.9% (1.9, 4.5)	X	X
**Regional prevalence**	**7.5% (5.9, 9.6)**	**6.1% (4.7, 7.8)**	**9.0% (7.2, 11.0)**	**5.6% (4.4, 7.1)**	**2.3% (1.5, 3.6)**	**2.6% (1.6, 4.1)**
**North Africa**						
Algeria (2011)	X	X	18.5% (15.1, 22.6)	1.3% (0.9, 2.1)	4.0% (2.6, 5.9)	0.4% (0.2, 1.0)
Egypt (2011)	X	X	8.4% (4.8, 14.4)	1.7% (0.8, 3.2)	X	X
Libya (2007)	X	X	6.7% (5.4, 8.3)	1.5% (0.8, 2.6)	X	X
Mauritania (2010)	X	X	17.9% (14.3, 22.1)	16.4% (11.9, 22.2)	7.0% (3.8, 12.5)	6.3% (3.2, 12.2)
Morocco (2016)	X	X	8.4% (7.1, 9.8)	2.8% (2.1, 3.7)	8.0% (6.2, 10.4)	2.2% (1.4, 3.4)
Tunisia (2008)	X	X	13.9% (10.7, 17.9)	3.0% (1.9, 4.8)	X	X
**Regional prevalence**	X	X	**9.2% (6.1, 13.7)**	**1.7% (1.0, 2.9)**	**5.7% (4.6, 7.1)**	**1.1% (0.8, 2.3)**
**Southern Africa**						
Botswana (2005)	24.1% (21.2, 27.2)	21.1% (18.0, 24.5)	10.9% (9.6, 12.4)	4.8% (3.6, 6.5)	X	X
Eswatini (2013)	X	X	X	X	5.2% (3.8, 7.1)	1.4% (0.8, 3.6)
Malawi (2009)	5.3% (2.9, 9.4)	2.4% (0.8, 6.4)	5.9% (4.0, 8.5)	3.5% (2.0, 6.2)	X	X
Mozambique (2015)	10.9% (7.4, 15.7)	10.6% (6.7, 16.4)	1.2% (0.5, 3.0)	2.9% (1.1, 7.4)	2.3% (1.0, 5.1)	0.5% (0.1, 2.4)
Namibia (2013)	29.7% (25.7, 34.1)	24.7% (22.8, 26.7)	12.0% (9.8, 14.7)	6.0% (4.2, 8.5)	6.8% (5.1, 9.0)	3.7% (2.3, 5.8)
Zambia (2004)	24.5% (20.8, 28.6)	28.9% (26.1, 31.8)	X	X	X	X
Zimbabwe (2003)	17.6% (14.6, 21.0)	10.9% (8.2, 14.4)	12.2% (10.3, 14.4)	7.4% (5.7, 9.5)	X	X
**Regional Prevalence**	**11.0% (8.4, 14.3)**	**10.4% (7.5, 14.2)**	**2.7% (1.8, 4.2)**	**3.2% (1.8, 5.9)**	**2.3% (1.1, 5.0)**	**0.6% (0.1, 2.3)**
**West Africa**						

Benin (2016)	40.9% (35.3, 46.8)	38.2% (32.9, 43.9)	5.6% (3.5, 8.9)	1.3% (0.6, 2.8)	1.3% (0.5, 3.3)	0.2% (0.0, 1.2)
Ghana (2012)	16.1% (13.8, 18.6)	13.1% (9.9, 17.2)	8.9% (6.4, 12.2)	7.8% (4.3, 13.6)	6.0% (4.5, 8.0)	8.4% (5.2, 13.2)
Liberia (2017)	21.6% (16.9, 27.1)	13.7% (10.3, 18.0)	7.1% (4.8, 10.4)	5.9% (4.2, 8.2)	7.2% (4.6, 11.2)	4.3% (2.3, 7.9)
Senegal (2005)	7.0% (3.8, 12.6)	4.0% (2.3, 6.8)	12.2% (6.5, 21.6)	3.2% (1.1, 9.3)	X	X
Sierra Leone (2017)	14.8% (10.7, 20.1)	8.6% (6.2, 11.8)	X	X	5.6% (3.5, 8.6)	2.8% (1.3, 5.8)
**Regional Prevalence**	**18.5% (15.3, 22.2)**	**14.0% (11.5, 16.9)**	**8.7% (6.9, 11.0)**	**6.8% (4.2, 10.8)**	**5.2% (3.8, 7.0)**	**7.4% (4.8, 11.2)**
**World Bank income group**						
Upper middle-income	26.9% (24.7, 29.3)	23.1% (21.1, 25.2)	7.4% (5.9, 9.2)	2.1% (1.4, 3.1)	7.1% (5.6, 9.0)	3.5% (2.2, 5.5)
Lower middle income	10.6% (9.3, 12.1)	8.2% (6.8, 9.9)	9.2% (7.0, 12.0)	3.4% (2.7, 4.4)	3.8% (3.1, 4.6)	3.3% (2.5, 4.5)
Low income	10.3% (8.0, 13.0)	8.6% (6.2, 11.8)	5.5% (4.2, 7.0)	3.6% (2.5, 5.3)	2.6% (1.3, 4.9)	0.7% (0.3, 2.0)
**Overall prevalence**	**10.6% (9.4, 11.9)**	**8.3% (7.1, 9.7)**	**8.6% (6.8, 11.0)**	**3.5% (2.8, 4.3)**	**3.6% (3.0, 4.4)**	**3.0% (2.2, 4.0)**

Notes: X indicates data unavailability

Supplementary Table 4. Prevalence of dual use of the substances by region, income group, and country

	Dual alcohol & cigarette use		Dual alcohol & marijuana use		Dual cigarette & marijuana use	
	**Boys (95% CI)**	**Girls (95% CI)**	**Boys (95% CI)**	**Girls (95% CI)**	**Boys (95% CI)**	**Girls (95% CI)**
**East Africa**						
Djibouti (2007)	X	X	X	X	X	X
Kenya (2003)	9.4% (5.4, 15.7)	6.7% (3.6, 12.1)	X	X	X	X
Mauritius (2017)	13.1% (10.5, 16.3)	6.9% (4.3, 10.9)	6.7% (5.4, 8.2)	1.1% (0.6, 2.0)	7.1% (5.5, 9.1)	1.1% (0.6, 1.8)
Seychelles (2015)	18.5% (15.6, 21.9)	13.6% (11.2, 16.4)	9.7% (7.6, 12.6)	4.1% (3.0, 5.7)	9.6% (7.6, 12.1)	3.6% (2.6, 5.0)
Sudan (2012)	X	X	X	X	X	X
Tanzania (2014)	1.4% (0.8, 2.7)	1.3% (0.7, 2.4)	1.1% (0.6, 1.9)	1.2% (0.7, 2.1)	0.6% (0.3, 1.2)	1.1% (0.6, 2.0)
Uganda (2003)	3.2% (2.4, 4.2)	1.4% (0.8, 2.3)	X	X	X	X
**Regional prevalence**	**3.4% (2.2, 5.1)**	**2.5% (1.6, 3.9)**	**1.2% (0.7, 2.0)**	**1.3% (0.7, 2.4)**	**0.6% (0.3, 1.2)**	**1.1% (0.6, 2.0)**
**North Africa**						
Algeria (2011)	X	X	X	X	2.5% (1.5, 4.0)	0.0% (0.0, 0.3)
Egypt (2011)	X	X	X	X	X	X
Libya (2007)	X	X	X	X	X	X
Mauritania (2010)	X	X	X	X	4.7% (2.7, 8.0)	4.5% (2.2, 9.0)
Morocco (2016)	X	X	X	X	3.3% (2.4, 4.4)	0.6% (0.3, 1.2)
Tunisia (2008)	X	X	X	X	X	X
**Regional prevalence**	X	X	X	X	**2.8% (2.0, 3.8)**	**0.3% (0.2, 0.5)**
**Southern Africa**						
Botswana (2005)	8.2% (6.3, 10.5)	3.7% (2.8, 5.0)	X	X	X	X
Eswatini (2013)	X	X	X	X	X	X
Malawi (2009)	2.2% (1.0, 4.9)	0.8% (0.3, 2.4)	X	X	X	X
Mozambique (2015)	0.7% (0.2, 2.1)	1.4% (0.5, 3.4)	2.0% (0.8, 5.3)	0.1% (0.0, 0.8)	0.7% (0.2, 2.3)	0.0%
Namibia (2013)	8.7% (6.6, 11.3)	4.1% (2.7, 6.3)	4.5% (3.3, 6.2)	2.2% (1.3, 3.5)	3.5% (2.4, 5.1)	2.1% (1.1, 3.7)
Zambia (2004)	X	X	X	X	X	X
Zimbabwe (2003)	7.0% (5.5, 8.8)	3.4% (2.4, 4.9)	X	X	X	X

**Regional Prevalence**	**1.3% 0.7, 2.3)**	**1.3% (0.6, 2.5)**	**2.1% (0.8, 5.1)**	**0.1% (0.0, 0.7)**	**0.7% (0.2, 2.3)**	**0.02% (0.0, 0.04)**
**West Africa**						
Benin (2016)	5.5% (3.5, 8.7)	1.5% (0.7, 3.2)	1.1% (0.4, 3.5)	0.2% (0.0, 1.4)	0.5% (0.1, 1.6)	0.2% (0.0, 1.3)
Ghana (2017)	5.2% (3.6, 7.3)	5.2% (3.0, 8.9)	4.6% (3.4, 6.3)	4.5% (2.9, 6.9)	3.0% (1.8, 5.0)	3.7% (2.1, 6.9)
Liberia (2008)	3.4% (1.6, 7.2)	2.5% (1.4, 4.6)	3.5% (2.1, 5.9)	1.8% (0.8, 4.1)	2.6% (1.2, 5.4)	2.3% (1.2, 4.2)
Senegal (2005)	4.5% (2.3, 8.7)	0.5% (0.2, 1.2)	X	X	X	X
Sierra Leone (2017)	X	X	X	X	X	X
**Regional Prevalence**	**5.1% (3.9, 6.7)**	**4.4% (2.6, 7.4)**	**4.0% (2.9, 5.6)**	**3.9% (2.6, 6.2)**	**2.5% (1.5, 4.2)**	**3.3% (1.9, 5.8)**
**World Bank income group**						
Upper middle-income	8.9% (7.4,10.4)	4.1% (3.1, 5.5)	4.8% (3.6, 6.4)	2.1% (1.3, 3.2)	3.9% (2.8, 5.5)	2.0% (1.1, 3.4)
Lower middle income	3.9% (3.0, 5.1)	3.0% (2.1, 4.3)	2.0% (1.5, 2.7)	2.1% (1.4, 3.1)	1.6% (1.2, 2.1)	1.4% (0.9, 2.0)
Low income	1.6% (1.1, 2.4)	1.2% (0.7, 2.3)	2.2% (0.9, 4.8)	0.2% (0.1, 0.6)	0.8% (0.3, 2.3)	0.1% (0.0, 0.2)
**Overall Prevalence**	**3.4% (2.6, 4.3)**	**2.7 (1.9, 3.7)**	**2.1 (1.5, 2.8)**	**1.8% (1.2, 2.6)**	**1.5 (1.1, 2.0)**	**1.2 (0.8, 1.8)**

Notes: X indicates data unavailability.

Supplementary Table 5. Sensitivity analysis (robustness checks) of the prevalence of different substances by gender, African region, and World Bank income group (2010 – 2017)

	Alcohol (95% CI)	Cigarettes (95% CI)	Marijuana (95% CI)	Dual alcohol & cigarettes (95% CI)	Dual alcohol & marijuana (95% CI)	Dual cigarettes & marijuana (95% CI)
**African_region**						
East Africa	4.6% (3.4, 6.1)	5.2% (4.1, 6.6)	2.6% (1.7, 4.0)	1.5% (0.9, 2.5)	1.4% (0.8, 2.3)	0.9% (0.6, 1.6)
North Africa	X	5.6% (3.8, 8.4)	3.5% (2.9, 4.3)	X	X	1.6% (1.2, 2.1)
Southern Africa	10.4% (6.9, 15.2)	2.2% (1.0, 5.0)	1.4% (0.7, 2.7)	1.0% (0.5, 1.9)	1.1% (0.4, 2.6)	0.4% (0.1, 1.1)
West Africa	17.6% (14.9, 20.7)	7.9% (5.7, 10.8)	6.4% (4.6, 8.8)	5.0% (3.6, 7.1)	4.0% (2.6, 5.6)	2.9% (1.9, 4.6)
**World Bank Income Group**						
Upper middle income	26.5% (23.8, 29.5)	11.5% (10.0, 13.1)	5.1% (3.9, 6.6)	6.5% (5.1, 8.3)	3.3% (2.5, 4.4)	2.8% (2.0, 4.0)
Lower middle income	8.3% (7.1, 9.8)	6.2% (5.2, 7.5)	3.7% (3.0, 4.6)	2.5% (1.9, 3.4)	2.1% (1.6, 2.9)	1.5% (1.2, 2.0)
Low income	10.5% (7.3, 15.0)	5.0% (3.8, 6.6)	2.6% (1.8, 3.6)	1.0% (0.6, 1.8)	1.2% (0.6, 4.4)	0.4% (0.2, 1.1)
**Gender**						
Boys	9.8% (8.3, 11.4)	7.9% (5.9, 10.6)	3.6% (3.0, 4.4)	2.3% (1.7, 3.0)	2.1% (1.5, 2.8)	1.5% (1.1, 2.0)
Girls	7.7% (6.4, 9.3)	2.8% (2.1, 3.7)	3.0% (2.1, 4.0)	2.1% (1.5, 3.1)	1.8% (1.2, 2.6)	1.2% (0.8, 1.)
**Overall prevalence**	**8.8% (7.5, 10.2)**	**5.5% (4.2, 7.2)**	**3.4% (2.7, 4.2)**	**2.3% (1.7, 3.0)**	**2.0% (1.4, 2.7)**	**1.4% (1.1, 1.8)**

Notes: X indicates data unavailability.
