# Supplementary material for: What are the factors associated with alcohol, cigarette and marijuana use among adolescents in Africa? Evidence from the Global School-based Health Survey
Source: BMJ Open. 2025 Jul 28;15(7):e089096. doi: 10.1136/bmjopen-2024-089096 (PMC12306306; doi:10.1136/bmjopen-2024-089096)
Supplement: Supplementary file 2 [file bmjopen-2024-089096.reviewer_comments.pdf]

PEER REVIEW HISTORY

BMJ Open publishes all reviews undertaken for accepted manuscripts. Reviewers are asked to complete a checklist review form and are provided with free text boxes to elaborate on their assessment. These free text comments are reproduced below.

ARTICLE DETAILS

Title (Provisional)

The prevalence and factors associated with alcohol, cigarette, and marijuana use among adolescents in 25 African countries: evidence from the Global School-based Health Surveys

Authors

Pokothoane, Retselisitsoe; Gelibo, Terefe Agerfa; Tsague, Josiane Djiofack; Mdege, Noreen Dadirai

---

VERSION 1 - REVIEW

**Reviewer**	**1**
**Name**	**Best, Catherine**
**Affiliation**	**University of Stirling, School of Health Sciences**
**Date**	**13-Jul-2024**
**COI**	**I have no competing interests.**

Review of bmjopen-2024-089096 The prevalence and factors associated with alcohol, cigarette, and marijuana use among adolescents in 25 African countries: evidence from the Global School-Based Health Surveys

Thank you for asking me to review this manuscript. The article is on an important topic.

The comments I have are as follows:

The main comment I have is that the survey weights, as stated in the methods section, ‘adjust the surveys for non -responses and sample representation in each country’ ie they are within country weights. They do not adjust for the differences in population between countries. When the datasets for each country are appended, I am not sure that a combined prevalence will take account of the different population sizes of each country. There are very significant differences in the populations of the included countries e.g. Kenya and Uganda are very roughly 50 million and Botswana roughly 2 and a half million. The GSHS samples for each country do not vary by a factor of 10. Given the country level prevalences for use of each of the substances vary markedly, a pooled prevalence would need to be weighted for population size. If this has been done, then this needs to be explained in the methods.

Without weighting at the country level, the overall prevalences (across all included countries) and the region-level prevalence estimates will be incorrect I believe.

The paper 'Fuhr, D. C., Fleischmann, A., Riley, L., Kann, L., & Poznyak, V. (2013). Alcohol and other psychoactive substances in Africa and the Americas: results from the WHO Global School-based Student Health Survey. Journal of Substance Use, 19(3), 274–282. https://doi.org/10.3109/14659891.2013.824038' presents some of the same data and should be referenced and the results compared and contrasted.

In the notes under the tables, there is a statement that the analysis has been adjusted for clustering of observations. I assume this is at the country level but this should be stated explicitly if this is the case.

**Reviewer**	**2**
**Name**	**Möckl, Justin**
**Affiliation**	**Institut für Therapieforschung**
**Date**	**16-Jul-2024**
**COI**	**None**

The paper presents the most recent prevalence estimates of use of alcohol, cigarettes, and marijuana, their dual use as well associated risk factors for students aged 11 to 16 years in Africa using the Global School-Based Health Surveys. The paper is well structured and written. The main critique would be the appropriateness of the estimates, keeping in mind the different times of the data collection starting in 2003 and ending in 2017. The authors try to evaluate the bias by excluding data from before 2010 in a sensitivity analysis and see no difference in the resulting estimates. Nevertheless, it is difficult to interpret the resulting prevalence since it encompasses different countries at different times, which makes the disentanglement between differences due to time or country impossible. However, this is not the authors' fault, as they strive to extract as much information as possible from the available data. This study highlights the need for more and newer data on this topic and analyzes the data available until now. I therefore think it can be an important study and a reference point for further studies with hopefully more recent data. In my opinion revising the method section to include more specific information about the analyses as well as a more in-depth discussion of the results would benefit the paper.

In the following, I have listed general remarks and detailed comments for each section of the manuscript.

General

1. Why have specific regions not asked questions about specific substances?

Abstract

2. Line 33/34. "The prevalence of cigarette smoking was significantly higher among boys than girls, but that of alcohol and marijuana was insignificant." It is not clear what was insignificant in the second part of the sentence. I guess the gender difference is meant, but I would clarify that. Also, I think there is one verb too much in the following sentence, since the listing of characteristics starts with "was associated" and ends with "were positively associated".
3. I agree with the conclusions but would appreciate an argument in the conclusions on the basis of the results presented to support them.

Introduction

4. Page 5, Line 11. Could you please add to what total population you are referring to in this sentence?
5. Page 5, Line 36 "Global School-Based Health Based Survey (GSHS)" I think there is a typo in the name of the study.
6. Page 5, Line 37 Is it possible to list the African countries that have already been analyzed in this sentence or add some information to get an idea, which regions and how many countries have been studied before?

Methods

7. Could you add which information was incorporated in the sample weights?
8. Page 6, Line 48: there is a typo. It should not say "any smoking day" since it is about alcohol.
9. Page 6, Line 56: I would suggest changing the phrase "at least once a month" because it can be understood that the prevalence is based on a frequency pattern of use although it is constructed similarly to alcohol and cigarettes.

Results

10. I feel that the different time points of the estimates are one main critique in the pooling of the prevalence, since you cannot disentangle the effect of region compared to survey year. This can be relevant as for example data in High-Income Countries is suggesting a general decline, even if there are regional differences to the timing and magnitude (Vashishtha et al., 2021). Have the authors tried to additionally include survey year in the logit model, to evaluate the impact of the different survey years on the model?
11. Page 9, Line 42 ff.: The sentence “The mean percentage of people living in the urban areas (urbanization) was 48.8% (sd:7.8%).” is easy to misunderstand, since this is not an individual but a country specific variable. The sentence can easily be understood as if the variable is an individual characteristic.
12. The variable of urbanization does not refer to the total sample of the study, e.g., share of urbanization for students in the specific age groups, but for the total population in the respective countries. Is there any reason to believe that the share of urbanization would differ concerning the total sample, which only consists of school children?
13. Page 10, Line 49: I think there is a grammatical error in the sentence. It should say “most common” instead of “more common”, right?
14. Figure 1: Why are the confidence intervals for Alcohol and Marijuana Use for Boys in the age of 11 negative? How are the confidence intervals constructed? I thought Stata is using a logit transformation to bind the CIs of proportions within 0 and 1.
15. How were statistical differences between gender by age groups calculated? Looking at figure 1 it seems that also for alcohol and Marijuana for older ages the confidence intervals are not overlapping, and therefore suggesting a significant difference. The gender gap is seemingly growing by age, which would be interesting, since data from the most recent Health Behaviour in School-aged Children study reports a closing gender gap by age in Europe, central Asia and Canada (Charrier et al., 2024). How would you explain these different findings?
16. Tables 3 to 4 are difficult to read, since there is too much space between the numbers and the first row overlaps with the numbering. I would suggest formatting the tables a little more compactly.

Discussion

17. In general, the discussion of the results mostly reads like a listing of results against current literature. The discussion would greatly benefit from arguments about why results may differ compared to other studies cited.
18. Page 19, Line 27: I don't see how the prevalence estimates presented suggest anything about developing higher chances for an addiction. There was no information about addiction in the analyses of the paper. Please revise this statement.

Literature

Vashishtha R, Pennay A, Dietze P, Barrientos Marzan M, Room R, Livingston M, Trends in adolescent drinking across 39 high-income countries: exploring the timing and magnitude of decline, *European Journal of Public Health*, Volume 31, Issue 2, April 2021, Pages 424–431, https://doi.org/10.1093/eurpub/ckaa193

Charrier L, van Dorsselaer S, Canale N, Baska T, Kilibarda B, Comoretto RI et al. A focus on adolescent substance use in Europe, central Asia and Canada. Health Behaviour in School-aged Children international report from the 2021/2022 survey. Volume 3. Copenhagen: WHO Regional Office for Europe; 2024. Licence: CC BY-NC-SA 3.0 IGO

VERSION 1 - AUTHOR RESPONSE

Reviewers' Comments	Authors' Responses
**Reviewer 1** – Dr Catherine Best, University of Stirling	
The main comment I have is that the survey weights, as stated in the methods section, 'adjust the surveys for non -responses and sample representation in each country' i.e., they are within country weights. They do not adjust for the differences in population between countries. When the datasets for each country are appended, I am not sure that a combined prevalence will take account of the different population sizes of each country. There are very significant differences in the populations of the included countries e.g. Kenya and Uganda are very roughly 50 million and Botswana roughly 2 and a half million.	We thank the reviewer for these insights, and have revised the weights appropriately. It's true, the between analysis needed re-weighting to take into account the population sizes of different countries, and samples.
- The GSHS samples for each country do not vary by a factor of 10. Given the country level prevalences for use of each of the substances vary markedly, a pooled prevalence would need to be weighted for population size. If this has been done, then this needs to be explained in the methods. - Without weighting at the country level, the overall prevalences (across all included countries) and the region-level prevalence estimates will be incorrect I believe.	We thank the reviewer for this important point. We have adjusted the weights appropriately. The pooled results as well as the regression results have indeed changed.

The paper 'Fuhr, D. C., Fleischmann, A., Riley, L., Kann, L., & Poznyak, V. (2013). Alcohol and other psychoactive substances in Africa and the Americas: results from the WHO Global School-based Student Health Survey. Journal of Substance Use, 19(3), 274–282. https://doi.org/10.3109/14659891.2013.824038' presents some of the same data and should be referenced and the results compared and contrasted.	
In the notes under the tables, there is a statement that the analysis has been adjusted for clustering of observations. I assume this is at the country level but this should be stated explicitly if this is the case.	Thank you for this. We have indeed clustered the standard errors at the country level. We have now stated this explicitly.
**Reviewer #2 - Dr. Justin Möckl, Institut für Therapieforschung**	
General<br><br>1. Why have specific regions not asked questions about specific substances?	We thank the reviewer for raising this issue. However, we are unable to answer this question as it is not clear from the GSHS questionnaires. Plausibly, this may be because at the time of the survey, some specific substances were not available in those particular countries/regions.
Abstract<br><br>2. Line 33/34. "The prevalence of cigarette smoking was significantly higher among boys than girls, but that of alcohol and marijuana was insignificant." It is not clear what was insignificant in the second part of the sentence. I guess the gender difference is meant, but I would clarify that. Also, I think there is one verb too much in the following sentence, since the listing of characteristics starts with "was associated" and ends with "were positively associated".	Many thanks. We have revised this for clarity.
3. I agree with the conclusions but would appreciate an argument in the conclusions on the basis of the results presented to support them.	Thank you for the comment. We have amended the conclusion to include recommendations based on the results .
Introduction<br><br>4. Page 5, Line 11. Could you please add to what total population you are referring to in this sentence?	We cannot see the part that refers to "total population", However, we have assumed that this is referring to the number of deaths given in the introduction. We

	have now indicated that these are worldwide/ global figures.
5. Page 5, Line 36 “Global School-Based Health Based Survey (GSHS)” I think there is a typo in the name of the study.	Many thanks for pointing out this. We have corrected the two typos in the abbreviation.
6. Page 5, Line 37 Is it possible to list the African countries that have already been analyzed in this sentence or add some information to get an idea, which regions and how many countries have been studied before?	It’s not possible to include these countries as there are a number of them (23), for readability purposes. We hope that, by providing the study references, if interested, the reader will be able to look these up. However, we provided the names of two additional countries, which have not been included in previous cross-country analysis. In addition, Table 1 (in the results section) lists all the countries included in the study.
Methods<br><br>7. Could you add which information was incorporated in the sample weights?<br><br>9. Page 6, Line 56: I would suggest changing the phrase “at least once a month” because it can be understood that the prevalence is based on a frequency pattern of use although it is constructed similarly to alcohol and cigarettes.	Thank you for this suggestion. We have clarified the sample weighting issues by providing more details in the data analysis section. We have also changed the phrasing.
8. Page 6, Line 48: there is a typo. It should not say “any smoking day” since it is about alcohol.	We thank the reviewer for catching this. We have corrected it.
Results<br><br>10. I feel that the different time points of the estimates are one main critique in the pooling of the prevalence, since you cannot disentangle the effect of region compared to survey year. This can be relevant as for example data in High-Income Countries is suggesting a general decline, even if there are regional differences to the timing and magnitude (Vashishtha et al., 2021). Have the authors tried to additionally include survey year in the logit model, to evaluate the impact of the different survey years on the model?	We have controlled for the survey years in all our regression models, but did not report the estimates because of space. Below the regression tables, there is a note “The survey year fixed effects have been included in all three regressions”, showing that we have accounted for different surveys.

	Wealso performed a sensitivity analysis where we excluded studies published before 2010 to confirm the robustness of our results.This is reported in the methods and results sections .
11. Page 9, Line 42 ff.: The sentence "The mean percentage of people living in the urban areas (urbanization) was 48.8% (sd: 7.8%)." is easy to misunderstand, since this is not an individual but a country specific variable. The sentence can easily be understood as if the variable is an individual characteristic.	We thank the reviewer for this. We have removed this interpretation.
12. The variable of urbanization does not refer to the total sample of the study, e.g., share of urbanization for students in the specific age groups, but for the total population in the respective countries. Is there any reason to believe that the share of urbanization would differ concerning the total sample, which only consists of school children?<br><br>13. Page 10, Line 49: I think there is a grammatical error in the sentence. It should say "most common" instead of "more common", right?	We have removed this variable from our regression analysis because of this misconception and the fact that it was highly correlated with location variables like region and income group and produced ambiguously high coefficients of the Odds Ratio.<br><br>This has been corrected.
14. Figure 1: Why are the confidence intervals for Alcohol and Marijuana Use for Boys in the age of 11 negative? How are the confidence intervals constructed? I thought Stata is using a logit transformation to bind the CIs of proportions within 0 and 1.	There was a typo. When plotting the bar graphs with confidence intervals, we used the Stata command, "cibar". We noticed that "cibar" command produces standard errors that are different from the Logit Model when computing the proportions. The different standard errors had an impact on the margin of errors and, hence, the intervals.<br><br>We have thus ensured that the standard errors that are used are from the Logit model and are computed using the Stata command "proportion". After ensuring that the point estimates and the standard errors are correct, we computed and graphed the

	confidence intervals manually to ensure that there were no errors.<br><br>We have now presented the revised graph that has confidence intervals that are within 0 and 1, as one would expect. We thank the reviewer for this important catch.
15. How were statistical differences between gender by age groups calculated? Looking at figure 1 it seems that also for alcohol and Marijuana for older ages the confidence intervals are not overlapping, and therefore suggesting a significant difference. The gender gap is seemingly growing by age, which would be interesting, since data from the most recent Health Behaviour in School-aged Children study reports a closing gender gap by age in Europe, central Asia and Canada (Charrier et al., 2024). How would you explain these different findings?	We thank the reviewer for these insights.<br><br>We have changed the graph type to clarify where overlaps are by gender- these show overlap, even for older ages for alcohol. For marijuana, it is only in the 16 years where there is no overlap.<br><br>We have incorporated your insights on the gender gap, how this is different from the observation in Charrier et al and why this might be so in the discussion section as follows (we hope this addresses your comment).<br><br>Our results also suggest a widening gender gap in current smoking status with increasing age between boys and girls. This is different from what has been observed in Europe, Central Asia and Canada, where data suggests no significant difference in current smoking between boys and girls in most countries and regions.22 This difference might be a reflection of differences in social acceptability of smoking among girls, with this being potentially higher in developed countries compared to Africa. For Africa, this, combined with the increased awareness, with age, that smoking is less acceptable for girls when compared to boys

	could mean that fewer girls take up smoking than boys as age increases.
16. Tables 3 to 4 are difficult to read, since there is too much space between the numbers and the first row overlaps with the numbering. I would suggest formatting the tables a little more compactly.	We have reduced the spacing between the columns and ensured that the first row (and other rows) do not overlap with the numbering when creating the pdf from the submission portal.
Discussion<br><br>17. In general, the discussion of the results mostly reads like a listing of results against current literature. The discussion would greatly benefit from arguments about why results may differ compared to other studies cited.	We have substantially revised the structure and content of this section and have provided potential arguments for different results. We thank the reviewer for this.
18. Page 19, Line 27: I don't see how the prevalence estimates presented suggest anything about developing higher chances for an addiction. There was no information about addiction in the analyses of the paper. Please revise this statement.	We are grateful for this. We have revised this statement.
Literature<br>Vashishtha R, Pennay A, Dietze P, Barrientos Marzan M, Room R, Livingston M, Trends in adolescent drinking across 39 high-income countries: exploring the timing and magnitude of decline, European Journal of Public Health, Volume 31, Issue 2, April 2021, Pages 424–431, https://doi.org/10.1093/eurpub/ckaa193<br><br>Charrier L, van Dorsselaer S, Canale N, Baska T, Kilibarda B, Comoretto RI et al. A focus on adolescent substance use in Europe, central Asia and Canada. Health Behaviour in School-aged Children international report from the 2021/2022 survey. Volume 3. Copenhagen: WHO Regional Office for Europe; 2024. Licence: CC BY-NC-SA 3.0 IGO	We thank the reviewer for sharing. Some have been useful to improve our discussion, especially policy insights on how the high-income countries have reduced high alcohol use among adolescents.

VERSION 2 - REVIEW

**Reviewer**	**1**
**Name**	**Best, Catherine**
**Affiliation**	**University of Stirling, School of Health Sciences**
**Date**	**14-Feb-2025**

COI

Review of 'The prevalence and factors associated with alcohol, cigarette, and marijuana use among adolescents in 25 African countries: evidence from the Global School-based Health Surveys'

Thank you to the authors for their clear responses.

1. A comment of mine that remains outstanding is that I noted that the paper 'Fuhr, D. C., Fleischmann, A., Riley, L., Kann, L., & Poznyak, V. (2013). Alcohol and other psychoactive substances in Africa and the Americas: results from the WHO Global School-based Student Health Survey. Journal of Substance Use, 19(3), 274–282. https://doi.org/10.3109/14659891.2013.824038' presents some of the same data and should be referenced and the results compared and contrasted. The Fuhr paper presents data from the GSHS from the same countries and presents data on the prevalence past 30 day smoking and alcohol use and the prevalence of dual use. I think it is important to reference this paper and comment on any differences in methodology (e.g. the Fuhr paper only reports data on 13-16 ages and the current paper used 11 and 12 year olds as well). The reference section in the revised tracked changes version of the paper under review, has no changes to the references from the original version and I think this may be an error as the references in the text have been edited so I think perhaps the authors intend to update the reference section as well and this has not come through in the resubmission process.

2. In table 2 some of the regional prevalences do not make sense in terms of the country level prevalences. For example for dual alcohol and marijuana use in East Africa there is data from Mauritius (3.6%) Seychelles (6.6%) and Tanzania (1.3%) and then the combined prevalence is reported as 0.6% which is lower than any of the constituent countries. This doesn't seem possible. The same is the case for dual cigarette and marijuana use in East Africa countries the prevalences are 3.8%, 6.2% and 0.9% then the combined East Africa regional prevalence is reported as 0.3% which again lower than all the countries for which there is data.

3. When comparing results to the study of 73 LMICs by de la Torre-Luque its worth noting that they used a different definition of 'use'.

4. When using the country level weights were these combined with the survey weights or instead of them?

5. In table 3 I take it that these variables are all the same model? If so, then the coefficients should be interpreted as mutually adjusted. That is, the effect of country religion is OR 0.9 on alcohol use after adjustment for income level, sex, parental smoking etc. See https://academic.oup.com/aje/article-abstract/177/4/292/147738?redirectedFrom=fulltext

VERSION 2 - AUTHOR RESPONSE

**Reviewers' Comments**	**Authors' Responses**
**Reviewer 1 –** Dr Catherine Best, University of Stirling	
A comment of mine that remains outstanding is that I noted that the paper 'Fuhr, D. C., Fleischmann, A., Riley, L., Kann, L., & Poznyak, V. (2013). Alcohol and other psychoactive substances in Africa and the Americas: results from the WHO Global School-based Student Health Survey. Journal of Substance Use, 19(3), 274–282. https://doi.org/10.3109/14659891.2013.824038' presents some of the same data and should be referenced and the results compared and contrasted. The Fuhr paper presents data from the GSHS from the same countries and presents data on the prevalence past 30 day smoking and alcohol use and the prevalence of dual use. I think it is important to reference this paper and comment on any differences in methodology (e.g. the Fuhr paper only reports data on 13-16 ages and the current paper used 11 and 12 year olds as well). The reference section in the revised tracked changes version of the paper under review, has no changes to the references from the original version and I think this may be an error as the references in the text have been edited so I think perhaps the authors intend to update the reference section as well and this has not come through in the resubmission process.	We appreciate this suggestion. We have now included the following text. "The other study closely related to ours examines the prevalence of cigarette smoking and alcohol use, utilizing GSHS data from nine countries in the African region and seven countries in the Americas for the years 2003 - 2007.[22] This study primarily focuses on comparing the country-level prevalences of cigarette use, alcohol consumption, and the dual use of cigarettes and alcohol. Among nine African countries, the prevalence of alcohol use ranged from 3.2% (Senegal) to 61.6% (Seychelles), and that of cigarette smoking from 3.8% (Tanzania) to 17.2% (Seychelles). The dual use prevalence of alcohol and cigarettes ranged from 1.5% (Tanzania) to 15.8 (Seychelles). These country-level prevalence results differ from our study mainly because we included students aged 11 to 16 years, while their study only included those aged 13 to 15 years.[22]<br><br>Additionally, their study[22] differs from ours in a number of ways. Unlike our study, they did not present pooled prevalences across countries, making it impossible to conduct pooled prevalence (overall and regional) comparisons. Their study[22] included 9 African GSHS datasets that were conducted up to 2007, while ours included additional 16 new datasets that were conducted up 2017. Finally, we also looked at the factors associated with the use of different substances, while their

	study[22] primarily focused on their prevalence of substance use."
In table 2 some of the regional prevalences do not make sense in terms of the country level prevalences. For example for dual alcohol and marijuana use in East Africa there is data from Mauritius (3.6%) Seychelles (6.6%) and Tanzania (1.3%) and then the combined prevalence is reported as 0.6% which is lower than any of the constituent countries. This doesn't seem possible. The same is the case for dual cigarette and marijuana use in East Africa countries the prevalences are 3.8%, 6.2% and 0.9% then the combined East Africa regional prevalence is reported as 0.3% which again lower than all the countries for which there is data.	Thank you very much for bringing this to our attention. We have corrected them.
When comparing results to the study of 73 LMICs by de la Torre-Luque its worth noting that they used a different definition of 'use'.	Thank you for noting this. We have added the following text: "The 73-country study used different measures of substance use from ours. For instance, while we focused only on current (past 30-day) use of the substances, that study focused on regular use and problematic use (especially for alcohol).[2]"
When using the country level weights were these combined with the survey weights or instead of them?	The survey weights provided with each country's survey were used to calculate the prevalence rates for each country without any adjustments. Further, for pooled analysis, we created a country-level weight based on sample sizes and populations of different countries,

	which was not combined with the survey weights.<br><br>However, in the current version we have combined (multiplied) these weights (survey and country-level weights) together. Nonetheless, this does not change the prevalence rates significantly.
In table 3, I take it that these variables are all the same model? If so, then the coefficients should be interpreted as mutually adjusted. That is, the effect of country religion is OR 0.9 on alcohol use after adjustment for income level, sex, parental smoking etc. See https://academic.oup.com/aje/article-abstract/177/4/292/147738?redirectedFrom=fulltext	Thank you for pointing out this. The coefficients are from the same regression model, and have been mutually adjusted. We have revised this in the write-up, and made it clear that these are actually adjusted Odds Ratios (aOR) in the Table and in the interpretations.
